# The Diversity Efforts Disparity in Academic Medicine

**DOI:** 10.3390/ijerph18094529

**Published:** 2021-04-24

**Authors:** Kendall M. Campbell

**Affiliations:** Director Research Group for Underrepresented Minorities in Academic Medicine, Brody School of Medicine, East Carolina University, Greenville, NC 27858, USA; campbellke16@ecu.edu

**Keywords:** diversity efforts disparity, academic medicine, underrepresented minority faculty

## Abstract

The diversity efforts disparity in academic medicine can be defined as part of the minority tax that negatively impacts faculty who are underrepresented in medicine. This disparity can be defined as differences between minority and non-minority faculty in their recruitment or assignment by the institution to address diversity issues, climate concerns and conflict around inclusion in academic medicine. It can manifest as disproportionate committee service, being asked to be the face of diversity for the school on websites or brochures or being asked to serve on diversity task forces or initiatives. In this article, the author further characterizes the diversity efforts disparity and provides recommendations for how to identify and address it in academic medicine.

## 1. Introduction

Faculty who are underrepresented minorities in medicine (URM) remain in low numbers in medical schools across the United States (US) [1]. This underrepresentation impacts the whole of academic medicine, from the classroom to the research lab to the exam room, and it also has implications for administrative roles in the institution. The limited contributions of URM faculty are evidenced in the fact that this group is in tenured and tenure earning tracks less often, even though in many instances, they have higher leadership aspirations than their white counterparts [2,3]. The more recent Rank Equity Index work by Fassiotto et al. also indicated that URM faculty are more heavily concentrated in lower academic ranks [4]. Because of limited faculty development, isolation, and limited mentorship, many leave academic medicine, sometimes only after five years on the job [5,6]. Groups who are URM include people who are Black or African American, Latinx, or Native American.

With few underrepresented minority faculty in academic medicine, opportunities for race-concordant mentoring are few for both peer mentoring and mentoring from leaders in the field [7,8]. Many times, faculty will seek sponsors more than mentors, and because of technology advances, long-distance mentoring and mosaic mentoring are a possibility. Cross-cultural mentoring plays a large roll for URM faculty and learners, as there are so few URM faculty that there are not enough to meet the URM mentoring need [9].

As these faculty members work through their careers, the literature has grown to provide more opportunities for academic leaders to understand and address the challenges that underrepresented minority faculty face [10,11]. These challenges have been long-lived and date back to the early 1900s with the report from Abraham Flexner, which led to the closing of several black medical schools [12,13]. The revolutionizing of medical education that came through his work was based on the racist idea that black doctors should only be sanitation doctors to keep illnesses that impacted minorities from impacting whites. Even as medicine has evolved, with URM physicians slowly increasing in numbers and in leadership, this thinking has persisted and perpetuated systems that advantage some and disadvantage others [14]. Even with the increases that we have seen in the absolute numbers, the numbers of black men in medicine lags, with more black men being admitted to medical school in the 1970s than in the 2000s [15].

In 2015, the minority tax was defined as it relates to the experiences of URM faculty in academic medicine [6]. The minority tax describes disparities or taxes that URM faculty face that impact their recruitment, advancement and retention. Those disparities include clinical efforts, promotion and diversity effort disparities, along with racism, lack of faculty development, lack of mentorship and isolation. The clinical efforts disparity describes how URM faculty are usually assigned more patient care duties and community service than their non-minority peers. Many times, this work is with underserved or poor populations that may not be insured, making it difficult for URM faculty to reach productivity and financial benchmarks established for them by their departments or by their employing hospital. Providing care for under-resourced communities can be difficult and stressful, due to having limited access to services and resources for care. The clinical efforts disparity leads to the promotion disparity, as the abundance of clinical work takes time away from the research and scholarship that are valued by the institution for promotion and tenure. When URM faculty have less time and support for developing ideas, writing papers and innovating in academic medicine, their ideas and talents may go unnoticed, and the medical school does not benefit. Not only that, but unnoticed contributions also trickle down to patient care and cause patients to lose out on inventions and innovations that could promote their health and improve clinical outcomes. Isolation is driven by URM faculty being excluded from relationships and opportunities. They are not part of the institutional culture and often feel like a poor fit [2]. A lack of faculty development and inadequate mentoring creates work environments that are not academically supportive for URM faculty, making it difficult for them to identify and harness the skills needed to be successful in their jobs and build sustainable and thriving careers in academic medicine. These activities are critical to URM faculty’s success and for creating pipelines to promotion in academic medicine. Faculty development opportunities that build skills and appreciate the uniqueness of cultural backgrounds and how they impact talent can be a benefit to URM faculty’s resilience and longevity [16].

In this paper, I choose to highlight the diversity efforts disparity of the minority tax. This disparity can be defined as differences between URM and non-URM faculty in their recruitment or assignment by the institution to address diversity issues, climate concerns and conflict around inclusion in academic medicine. The disparity can manifest as disproportionate committee service, being asked to be the face of diversity for the school on websites or brochures or being asked to serve on diversity task forces or initiatives with or without supported time, mentorship or resources, in addition to not having that service valued for promotion or tenure [3,6,17]. This burden can impact the delivery of clinical care to vulnerable populations, research and education, and it is a manifestation of the minority tax. Diversity efforts have been a highlighted need that has increased in recent years, as more US medical schools are looking to onboard a chief diversity officer or dean of diversity. Even positions of this nature, when appointed by university leadership, can serve as a diversity efforts disparity, because those appointed may not receive the office infrastructure or staff support to manage this work comparable to other dean level administrative offices. Diversity work is complicated, as many diversity and inclusion leaders are called to address climate and inclusion concerns that they did not create but that directly impact their existence at the institution because of URM status or similar backgrounds. Because URM in the US tend to go into primary care specialties more often [18,19], primary care specialties and academic centers have to be aware of and address this disparity. Moreover, in this current time of national response to the murder of George Floyd, the Black Lives Matter movement and the pre-election insurrection at the capital, recognizing the impact of the diversity efforts disparity during this time carries additional significance. Nationwide events can bring out allies, cause the development of support infrastructure and carry the message of injustice to a wider audience. This is what we have witnessed with the killing of unarmed black men and others by police. We have seen a response erupt from all over the world, with a movement and a spark of initiatives to further characterize and uproot anti-black racism in the US [20]. The national response has prompted discussions in medical schools all over the US to evaluate their curricula, policies, staffing and environments for racist practices and systems that advantage some and disadvantage others. That can translate into more diversity work and greater diversity pressures for URM faculty as medical school leaders go to this group more often for recommendations, solutions and initiatives.

When added along with racism, a lack of mentorship, lack of faculty development, promotion disparities and clinical efforts disparities, the diversity efforts disparity plays a more significant role in URM faculty leaving academic medicine [6,7]. Other aspects of the minority tax are discussed in greater detail elsewhere in the literature [6,10]. The diversity efforts disparity is less talked about and may not be as apparent because academic leaders are not conscious of its existence. Many times, URM faculty want to help with diversity and inclusion efforts because they want to see others who look like them at the institution, and they want to work in an inclusive work environment. Because of the continued low numbers of URM in academic health systems [21,22], efforts to retain this group, which include addressing the diversity efforts disparity, need continued exploration and support.

The diversity efforts disparity can become a source of fatigue, stress and frustration for a URM faculty member. Minority faculty have to balance the demands of their work role across educational, research and clinical responsibilities, along with the institutional pull of diversity responsibilities with the net of challenges URM faculty face [2,10]. In the face of limited faculty development and limited mentorship for URM faculty [6,8], efforts to address the impact of the diversity efforts disparity need to be investigated. This article provides recommendations for academic leaders on how to identify and address this disparity and how addressing this disparity impacts URM mentorship and leadership development in academic medicine.

Addressing the diversity efforts disparity has the potential to improve the experience of URM faculty in our academic institutions, and recognizing its existence is the first step in addressing inequitable diversity efforts. Increasing diversity in US medical schools among leaders to include deans and department chairs can increase diversity of thought and provide added perspective in leadership circles that can begin to address the disparity [23]. There are programs to increase the diversity of medical students, with some beginning at the pre-college level, but there are few to increase diversity of faculty aside from search committees [24,25]. After recognition by institutional leaders as a problem, the next stop for further characterizing and moving toward solutions is with the department of human resources. Leaders need to meet with their human resources department to review the racial and ethnic composition of all departments. Departmental leaders need to be held accountable for inattention to the diversity and inclusion needs of the institution and their individual areas of responsibility. They should also be held accountable for actions taken or initiatives created to increase diversity and promote inclusion in their departments. This accountability can be in the form of annual evaluation requirements or links to departmental incentives or funding. Department chairs can meet with diversity leaders of the institution to better define approaches to increase diversity among faculty and to identify non-URM faculty who want to champion this work. Leaders need to be clear on the hiring practices for recruitment diversity, such as ensuring the diversity of search committees and advertising in places where URM faculty are likely to see ads, such as minority-serving medical societies or minority-focused journals. In addition, when URM candidates are invited for interviews, it is important that candidates get a chance to see and interview with URM employees, as this may help address the isolation this group experiences in academic medicine [6].

In addition, just like knowing departmental diversity representation, it is important for leaders to know how many committees exist across the institution, along with the racial or ethnic composition of the members on those committees. This work is needed to determine if the density of URM faculty, learners and staff is out of proportion to their representation at the institution. If this is the case, or if it is found that the same URM sit on multiple committees, it should raise concern for the diversity efforts disparity. Committee diversity concentrated at the staff and student level—more so than the administrative and leadership level—is another indication that the diversity efforts disparity may be in play. As one ascends the leadership ladder in the institution, there is usually less and less racial and ethnic diversity [3]. Leaders have to be intentional about ensuring diversity throughout all levels of the institution. Additionally, leaders have to be aware of faculty burnout, as it can signal a diversity efforts disparity, particularly when burnout is more concentrated in URM faculty. It is important to address the causes of burnout, as burnout may lead to health concerns, low work performance and departure from academic medicine.

If there are few recruitment, mentoring or faculty development opportunities for URM faculty growth, that may mean there is not a pathway for URM faculty advancement at the institution, and the same URM faculty may be overused in committee work. Increased committee work, just like disproportional clinical work, can mean less time for scholarship, which translates to the promotion disparities that we often see for URM faculty in academic medicine [6]. To combat this concern, institutions should develop pathways for advancement for URM faculty that not only involve supporting their success through resources and staffing, but also dismantling the privilege systems and racism that hinder URM faculty success [8,26]. Institutions need to be intentional about creating URM deans, department chairs and division leaders and engaging internal and external resources to promote URM successes across the institution.

Academic leaders should know the demographics of their institution compared to that of their local community. It is important that the makeup of employees in medical schools reflects the communities in which they live. If the community is more diverse than the medical school, then there is increased risk of a diversity efforts disparity. Institutions need to utilize recruitment opportunities to increase the racial and ethnic diversity of their leadership, faculty and learners.

Fixing the diversity efforts disparity can impact the development and growth of URM mentors and leaders. Because there are few mentors for URM faculty who share similar cultures and backgrounds, strategies to increase these numbers are very important. The literature is clear on needing more mentors and the benefits of mentorship for faculty in academic medicine [27]. Developmental networks have the potential to assist a URM faculty member who is suffering from the diversity efforts disparity in that these networks have the ability to develop leadership [28]. These networks can combat isolation and promote inclusive leadership, creating an environment that includes all [29]. Growth in leadership and mentorship ability among URM faculty can create resilience to help overcome the diversity efforts disparity. A model to suggest a platform for this includes recent work by Coe et al., which explored pathways for leadership for URM family medicine faculty [30]. Although this study focused only on the primary care specialty of family medicine, their work is generalizable for URM faculty across specialties. Exploring pathways to leadership along with faculty development programming that is not just skills-based, but also includes URM faculty learning about the minority tax and other systems designed to advantage some and disadvantage others, can be a powerful tool to promote the success and advancement of this group [5,31]. In addition, sponsorship can be valuable to the success of URM faculty. When white colleagues open doors and provide opportunities for URM exposure and success, they promote the talents and development of URM faculty success [26].

## 2. Conclusions

In this paper, I have provided greater detail on how the diversity efforts disparity impacts URM faculty, along with recommendations to address it in US medical schools. The first is recognizing the problem exists. Further recommendations include determining the racial or ethnic compositions of departments in the institution to define the representation of URM faculty. Low representation can mean few URM faculty and the presence of a diversity efforts disparity. The number of URM faculty should be increased. Determine the racial or ethnic composition of committee membership to determine if there is overrepresentation of URM faculty on committees. If there is, decrease the number of URM faculty and recruit other faculty to serve. In addition, evaluate the ranks and hierarchy of committee members to ensure URM leaders are present and not just URM at the staff or student level. Senior leaders have to be held accountable to address this disparity, and non-URM allies have to be identified to share in this work and make it equitable. Leaders also have to develop pathways for advancement for URM faculty. Medical schools also need to know the demographics of the medical school compared to the local community and, if more URM are in the community, address the diversity efforts disparity by increasing diversity in the medical school. With lessons learned from URM faculty experiences and a growing body of literature available to assist academic leaders, we can dismantle this disparity and promote an environment of inclusion and success for this group in academic medicine and beyond.
